# Distribution of *Legionella pneumophila* bacteria and *Naegleria* and *Hartmannella* amoebae in thermal saline baths used in balneotherapy

**DOI:** 10.1007/s00436-012-3106-4

**Published:** 2012-09-30

**Authors:** Elżbieta Żbikowska, Maciej Walczak, Arkadiusz Krawiec

**Affiliations:** 1 Department of Invertebrate Zoology, Faculty of Biology and Earth Sciences, Nicolaus Copernicus University, Toruń, Poland; 2 Department of Water Microbiology and Biotechnology, Faculty of Biology and Earth Sciences, Nicolaus Copernicus University, Toruń, Poland; 3 Department of Geology and Hydrogeology, Faculty of Biology and Earth Sciences, Nicolaus Copernicus University, Toruń, Poland

## Abstract

The present study was aimed at investigating the coexistence and interactions between free living amoebae of *Naegleria* and *Hartmannella* genera and pathogenic *Legionella pneumophila* bacteria in thermal saline baths used in balneotherapy in central Poland. Water samples were collected from November 2010 to May 2011 at intervals longer than 1 month. The microorganisms were detected with the use of a very sensitive fluorescence in situ hybridisation method. In addition, the morphology of the amoebae was studied. Despite relatively high salinity level, ranging from 1.5 to 5.0 %, *L*. *pneumophila* were found in all investigated baths, although their number never exceeded 10^6^ cells dm^−3^. *Hartmannella* were not detected, while *Naegleria fowleri* were found in one bath. The observation that *N*. *fowleri* and *L*. *pneumophila* may coexist in thermal saline baths is the first observation emphasising potential threat from these microorganisms in balneotherapy.

## Introduction

First identified in 1976 (Saint and Ho 1999; Huang et al. 2011), *Legionella* sp. bacteria are one of the main groups of pathogenic bacteria transmitted via water (Papciak and Zamorska 2005) and belong to the gamma Proteobacteria (Heuner and Steinert 2003). Approximately half of 48 species of *Legionella* cause a disease called legionellosis (Legionnaires’ disease), most notably *Legionella pneumophila*, responsible for more than 80 % of all known cases (Yaňez et al. 2005). Human infection by *L*. *pneumophila* usually results from inhaling aerosol droplets of water, which contain bacterial cells (Turetgen et al. 2005).

Free living amoebae (FLA), including *Naegleria fowleri*, *Acanthamoeba* sp., *Balamuthia mandrillaris*, *Sappinia* sp. and *Hartmannella* sp., responsible for dangerous infections in humans and animals, enter the human body in a similar way (Martinez 1996; Schuster and Visvesvara 2004; Dykova and Lom 2004; Daft et al. 2005; Karanis et al. 2007; Visvesvara et al. 2005, 2007). *N*. *fowleri* causes primary amoebic meningoencephalitis, a disease of the central nervous system, resulting in death of the infected people (Song et al. 2008; Edagawa et al. 2009; Jamerson et al. 2009).

FLA, also referred to as deadly amoebae or “brain-eating” amoebae inhabit natural and anthropogenic aquatic environments (Behets et al. 2007; Schuster and Visvesvara 2004; Sheehan et al. 2003a; De Jonckheere 2002; Tyndall et al. 1989). The relationship between pathogenic species of *Legionella* and FLA has a unique character. *Legionella* sp. are parasites of *Naegleria* and *Hartmannella* amoebae, within which they multiply, acquiring new ways of spreading in the environment (Fields et al. 2002; Molmeret et al. 2008; Fields 1996; Abu Kwaik 1998; Heuner and Steinert, 2003; Ettinger et al. 2003; Huang et al. 2011). The above-mentioned ubiquitous species of FLA are frequently found in ecosystems contaminated with *Legionella* (Grimm et al. 2001; Greub and Raoult 2004; Suzan-Monti et al. 2006), providing food and shelter for the pathogenic bacteria. Owing to the fact that a range of other pathogenic organisms including *Mycobacterium avium*, viruses and fungi may also develop inside FLA, in scientific literature, they are referred to as “Trojan horses” (Edgawa et al. 2009) and represent a potential source of pathogens dangerous for humans (Horn and Wagner 2004).

Successful coexistence of *Legionella* and FLA is based mainly on their shared ability to grow in biofilms which form at the solid–liquid interfaces or at the liquid–air interface (Flemming et al. 2000; Huws et al. 2005; Hoffman and Michel 2001) as well as on their tolerance to elevated temperatures. *L*. *pneumophila* can survive in water at temperatures ranging from 0 to 70 °C, and their optimum temperature (32–35 °C) (Declerck et al. 2007) partially overlaps with the temperature conditions preferred by *Naegleria* and *Hartmannella* amoebae (30–42 °C) (Jamerson et al. 2009; Mazur 1984; Lorenzo-Morales et al. 2007; Pelandakis et al. 2000). Both the pathogenic bacteria and their host amoebae, which display tolerance to elevated temperatures, find favourable conditions for growth not only in water bodies in regions characterised by hot climate but also in heated water of baths, pools and other facilities used in recreation and balneotherapy. Taking into consideration the high pathogenicity of *Legionella* and several FLA amoebae, the study focused on establishing whether *L*. *pneumophila* can coexist with *Naegleria* and *Hartmannella* amoebae in thermal saline baths used in balneotherapy.

## Materials and methods

### The object of the investigation

Water samples were collected from thermal saline baths, supplied with thermal saline waters (type Cl–Na), containing mainly iodides and iron, components with pharmacodynamic properties (Table 1). Found at great depths (700–1,700 m), they are well isolated from surface waters and appear to contain almost no organic compounds. The temperature of the water in the intake ranges from 32 to 40 °C. There is a continuous water flow from the intake into the pipes.

**Table 1:** Physical and chemical properties of thermal saline waters

Components/properties	Intake 1, water for baths 1 and 2		Intake 2, water for bath 3	
	(mg dm^−3^)	(% milivals)	(mg dm^−3^)	(% milivals)
Mineralisation	43,520.0	–	76,164.0	–
pH	6.9	–	6.2	–
Na^+^	14,700.0	85.25	25,520.0	84.13
Ca^2+^	1,322.6	8.79	2,404.8	9.10
Mg^2+^	486.1	5.33	960.05	5.99
K^+^	163.2	0.01	196.6	0.38
Fe^2+^	1.25	0.01	10.5	0.03
Cl^−^	26,233.0	98.93	46,085.0	98.90
SO_4_^−^	96.7	0.28	510.26	0.81
HCO_3_^−^	355.8	0.78	187.2	0.23
Br^−^	7.4	0.01	98.0	0.14
J^−^	2.1	0.00	3.5	0.00
S H_2_S + HS^−^	0.90	–	ND	–
Temperature (°C)	28	–	40.5	–

*ND* no data

### Sampling

Water samples were collected from November 2010 to May 2011 (five sampling cycles) from three thermal baths: bath 1, water salinity 5 %; bath 2, water salinity 4 %; bath 3, water salinity 1.5 %. Baths 1 and 2 are used for balneotherapy; bath 3 is used for recreation only.

Each sampling operation involved collecting one litre of “open water” taken from the bath into sterile glass bottles from each bath and measuring the following physicochemical parameters of water: its temperature, redox potential, pH value (with the Elmetron pH meter) and oxygen saturation (with the Hanna Instruments oximetre). The samples were then transported to the laboratory in 7 °C.

### Fluorescence in situ hybridisation method

The numbers of bacteria belonging to different phylogenetic groups (*Eubacteria*, *Legionella* sp. and *L*. *pneumophila*) and the investigated eukaryotic organisms (Eukariota, *Naegleria* sp., *N*. *fowleri* and *Hartmannella* sp.) were determined with the use of a molecular fluorescence in situ hybridisation (FISH) method. After the end of water uptake, the water samples were fixed with formamide. Later, the water samples were filtered through polycarbonate membrane filters with a 0.22-μm pore size in order to capture particles bigger than 0.22 μm. The hybridisation was performed according to Grimm et al. 2001. Bacterial cells retained on the surface of the membrane were hybridised using *Legionella*-specific fluorescence-labelled (with dye CY3) oligonucleotide probes EUB338, LEG705, LEGPNE1 and fluorescein-labelled, eukaryote-specific probes EUK516, HART498, NAEG1088 and NAE1041.

The probes were suspended in the hybridisation buffer consisting of formamide [whose concentration depended on the probe sequence (in volumes per volume)], NaCl 0.9 M, sodium dodecyl sulphate 0.01 % and Tris/HCl (20 mM), pH 7.6. This solution was applied to the surface of the filter with captured cells.

For probes LEG705, LEGPNE1 and EUK516, formamide concentration was 25 %; for probes NAEG1088 and NAE1041, formamide concentration was 30 %; for probe EUB338, formamide concentration was 35 %, and for probe HART498, formamide concentration was 40 %. The probe concentration in the hybridisation buffer was 30 ng of probes for prokaryotic organisms and 150 ng of probes for eukaryotic organisms.

The filters were then placed for 2 h in a hybridisation chamber and in ultrathermostat at 46 °C. After that, in order to remove the unbound probes, the filters were placed for 15 min in the washing buffer having the temperature of 48 °C (the composition of the buffer, 20 mM Tris/HCl, pH 7.6; 0.01 % sodium dodecyl sulphate; 5 mM ethylene diamine triacetic acid (EDTA)-160 mM NaCl for probes LEG705, LEGPNE1 and EUK516; 5 mM EDTA; 56 mM NaCl for probe HART498; 5 mM EDTA; 112 mM NaCl for probes NAEG1088 and NAE1041; 80 mM NaCl for probe EUB338), rinsed with the distilled water and dried.

Subsequently, the filters were covered with the mixture of immersion oil and Citifluor AF2 (Citifluor Ltd., London, UK). Fluorescence was detected using Olympus BX50 microscope equipped for epifluorescence microscopy with a 50-W mercury high-pressure bulb and the appropriate filter set 00 and 10. The slides with hybridised prokaryotic cells were analysed under the lens at × 100 magnification, while the slides with eukaryotic cells were analysed at × 10 and 40 magnifications. Colour micrographs were taken with digital image processing (Olympus XC50) using the software package (Cell^B^ v. 3.1.). The number of bacteria in the investigated slides was evaluated using MultiScan-Based programme.

The images of hybridised amoebae were additionally evaluated for morphological features; identification of the size and morphological features typical of amoebae of *Naegleria* and *Hartmannella* genera was particularly significant. *Naegleria* sp. are quite uniform in shape—a cell reaches 10–15 μm. The cytoplasm is slightly granular and has a clearly visible bright halo with a dense nucleus. Numerous vacuoles are usually seen in the cytoplasm. Trophozoites move by extending and contracting their rounded pseudopodium (lobopodium), which are bright on the edges and filled with granular cytoplasm. The posterior end of the cell, a hyaline uroid, has many small pseudopodia.

The trophozoites of *Hartmannella* reach the size of 25–40 μm, have an elongated shape and produce monopodial lobopodia. In the cytoplasm of these amoebae, there are numerous bright areas corresponding to vacuolar vesicles. Due to the morphological similarity of pathogenic species of *Naegleria* and *Hartmannella* to non-pathogenic species as well as to small but real risk of non-specific binding of the probes to the particles of organic matter after hybridisation, only the combination of the two methods, i.e. molecular (FISH) and morphological, provides an accurate evaluation of the studied FLA. The number of amoebae detected and identified by fluorescence in situ hybridisation and confirmed in the morphological analysis was recalculated per 1 dm^3^ of the sampled water with the use of the above-mentioned formula.

## Results

The temperature in the investigated baths ranged from 30 to 36.5 °C (Table 2), providing optimum conditions for the growth of both *L*. *pneumophila* and *Naegleria* and *Hartmannella* amoebae. Slight fluctuations of pH values did not affect the growth of the studied microorganisms. However, oxygen saturation varied significantly; the biggest differences between consecutive measurements being recorded in thermal bath 1 (range 19.8–138 %).

**Table 2:** Physicochemical parameters of water in the investigated baths

Data of sampling	Bath 1			Bath 2			Bath 3		
	Temperature (°C)	pH	Oxygen (%)	Temperature (°C)	pH	Oxygen (%)	Temperature (°C)	pH	Oxygen (%)
11.2010	32.5	8.0	19.8	32.3	7.4	102.6	36.5	7.2	133.6
01.2011	32.3	7.3	138.0	32.4	7.4	119.0	36.1	7.3	91.7
02.2011	31.5	7.2	70.0	32.2	7.4	110.0	35.6	7.4	102.5
03.2011	30.7	7.5	107.3	32.2	7.4	102.0	32.5	7.4	85.0
05.2011	31.7	7.1	67.0	32.3	7.5	102.6	34.7	6.7	117.5

**Fig. 1:**
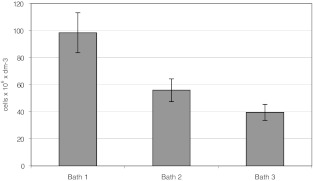
Number of *Eubacteria* in the investigated baths. *Vertical bars* represent standard deviation

The highest number of *Eubacteria* was recorded in bath 1. The average number in the entire research cycle for this bath was 98 × 10^6^ dm^−3^ cells. The lowest number of *Eubacteria* was recorded in bath 3 (Fig. 1). The average number in the entire research cycle for this bath was 39.5 × 10^6^ dm^−3^ cells.

Statistical analysis revealed no significant relationships between physicochemical parameters of water in the investigated baths and the number of *Eubacteria* (*P* > 0.05). In addition, no significant changes in the number of bacteria of the investigated group were recorded in particular baths in consecutive samples. Differences in the number of *Eubacteria* recorded in different baths, although substantial, were not significant statistically either (*P* > 0.05). The figures reveal positive correlation (*r* = 0.94) between the number of bacteria and the age of the bath.

**Fig. 2:**
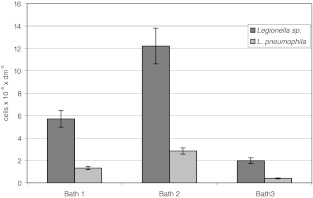
Number of *Legionella* sp. and *L*. *pneumophila* in the investigated baths. *Vertical bars* represent standard deviation

**Fig. 3:**
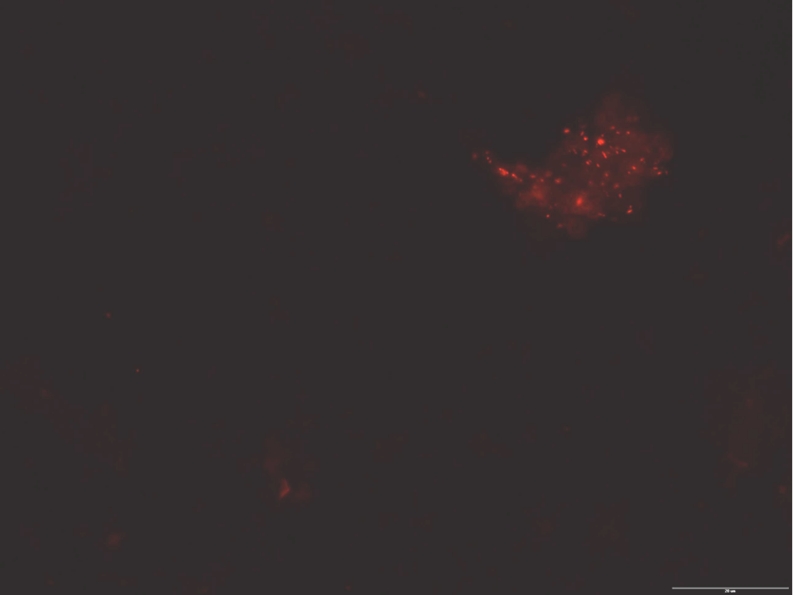
*L*. *pneumophila* bacteria in the fragment of biofilm in investigated sample water from bath 1, bar = 20 μm

Bacteria belonging to the *Legionella* sp. and *L*. *pneumophila* were identified in all water samples collected from each bath (Fig. 2). Moreover, in the investigated microscopic slides, *L*. *pneumophila* were also detected within the biofilm and microcosm (Fig. 3).

The highest number of bacteria belonging to the *Legionella* genus was recorded in bath 2 (12.21 × 10^6^ dm^−3^ on average). Furthermore, *L*. *pneumophila* were also the most abundant in bath 2, where their average number was 2.84 × 10^6^ dm^−3^ on average (Fig. 2).

The lowest number of *Legionella* sp. (1.98 × 10^6^ dm^−3^ on average) was observed in bath 3. *L*. *pneumophila* were also the least abundant in bath 3, where their average number was 0.40 × 10^6^ dm^−3^. In bath 1, the average number of bacteria of the *Legionella* genus was 5.71 × 10^6^ dm^−3^, while the average number of *L*. *pneumophila* was 2.84 × 106 dm^−3^.

**Fig. 4:**
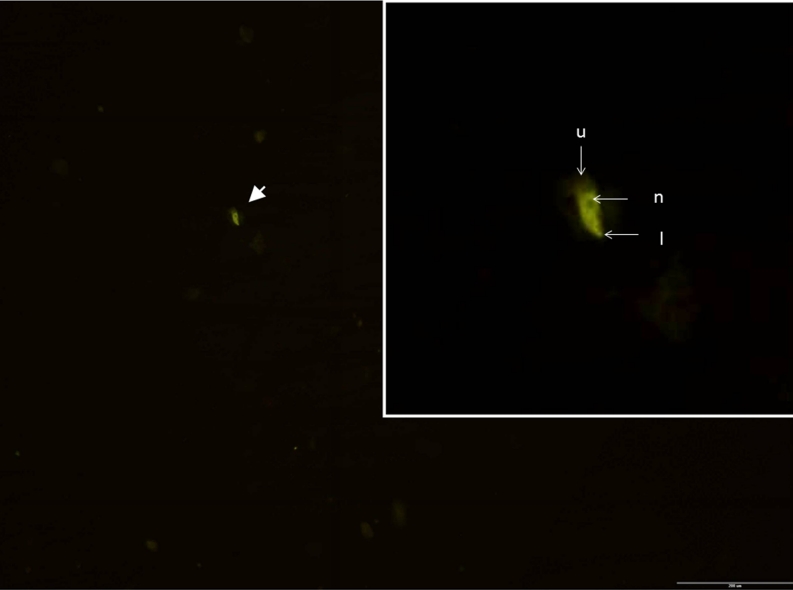
Positive result of FISH hybridisation for *N*. *fowleri*, bar = 200 μm. Amoeba is marked with an *arrow*: *l* lobopodium, *n* nucleus, *u* uroid. *Enlargement in* *a frame* indicates sample 1 from bath 1

Amoebae of the *Hartmannella* genus were not identified in any of the water samples taken from all baths. However, limax amoeba of *N*. *fowleri* sp. was identified in one sample taken from bath 1, based on the results of the FISH method (Fig. 4). Morphological features of the hybridised amoeba, including its length (15 μm), monopodial lobopodium, a hyaline uroid and a large kariosom in the nucleus, confirm its affiliation to *N*. *fowleri*.

## Discussion

In the hitherto published research papers, hardly any information is available on the distribution of *Legionella* sp. and FLA in thermal saline waters applied in balneotherapy. A majority of studies are aimed at identifying Legionellaceae in hot springs and in recreational facilities connected with the springs as well as in drinking and industrial water supply systems (water cooling systems, hydro-electric power stations). The results obtained during this investigation can be therefore regarded as original and innovative.

Taking into consideration the substantial salinity of the investigated thermal saline baths, the number of *L*. *pneumophila* seems to be large. However, Barbaree et al. (1983) observed that *L*. *pneumophila* serogroups 4 and 5 display the highest resistance to chloride sodium salt of all bacteria belonging to the genus *Legionella* sp. Besides, Palmer et al. (1993) confirmed the presence of *Legionella* sp. in 50 % of the collected samples of ocean water and the presence of *L*. *pneumophila* in 75 % of the samples.

Although the data suggest a fairly high tolerance of this kind of bacteria to high concentration of NaCl, it should be noted that all attempts to culture *Legionella* sp. from ocean water proved unsuccessful, which may indicate that only non-culturable cells of these microorganisms are present in saline waters. What is more, *Legionella* growth is the most rapid within the existing biofilm (Declerck 2010). Since biofilm is known to possess some properties protecting bacterial cells found deep within this formation, professional antiseptics have little or no effect on them. It may be therefore assumed that biofilm effectively protects *Legionella* against salt.

In this study, the number of *Legionella* fell within the range 10^3^ to 10^8^ CFU/dm^3^, which corresponds to the range observed in the natural environment by Stypułkowska-Misiurkiewicz et al. (2001). The results of the investigations conducted in hospitals and spas in Poland (including those focused on examining water from thermal saline baths) show that culturable forms of *L*. *pneumophila* were found in 78.7 % of the samples (Matuszewska and Krogulska 2009). In addition, in a majority of positive tests (71.7 % of the samples), the numbers of *L*. *pneumophila* were similar to the results obtained in this investigation and ranged from 1.2 × 10^2^ to 1.3 × 10^5^ cfu/100 ml.

Many researchers who applied a real-time PCR method to detect *Legionella* sp. and *L*. *pneumophila* emphasised the fact that these microorganisms are commonly found in anthropogenic water bodies. In their investigations, the presence of *Legionella* sp. and *L*. *pneumophila* was confirmed in all water samples collected from anthropogenic water bodies (Declerck et al. 2007). Huang et al. (2010) also found *Legionella* sp. in all recreational areas and facilities connected with hot springs in Taiwan. Twenty samples out of 72 collected in the research cycle contained *Legionella* sp. Considering the prevalence of these bacteria in the environments surrounding humans, a question should be answered whether the epidemics caused by *L*. *pneumophila* is a real threat.

According to Exner and Hartemann (2009), an acceptable number of *L*. *pneumophila* fall within the range 10^5^–10^6^ cfu/dm^3^, which may indicate that waters investigated in this study were relatively safe for humans. It must be stressed, however, that there were cases of legionellosis caused by *Legionella* in water systems when their number was just above 10^4^ cfu/dm^3^ (Meenhorst et al. 1985; Patterson et al. 1994).

In this study, water samples were also analysed for the presence of amoebae of *Naegleria* spp. and *Hartmannella* spp. *N*. *fowleri* was identified in one sample (taken from bath 1) (photo 8). Physicochemical and biotic conditions in the investigated baths deviate substantially from the conditions prevailing in the environments inhabited by *N*. *fowleri* and may therefore differently (contrarily) affect the growth of these amoebae (Sheehan et al. 2003b; Init et al. 2010). Amoebae are frequently found in fresh water, even strongly acidic or chlorinated (Rivera et al. 1983).

Osmotic pressure of the environment is one of the factors determining the differentiation of *Naegleria* amoebae into non-pathogenic flagellated forms (Jeffery and Hawkins 1976; Fulton 2004). Although high temperature, high oxygen saturation (Table 1) and a high number of bacteria (Fig. 1) in the investigated baths constitute favourable conditions for the development of amoebae, high salinity may be a limiting factor, preventing amoebae from rapid spread. Nevertheless, contrary to repeated published information suggesting the absence of pathogenic *Naegleria* in the marine environment, our observations agree with the observations of Culbertson (1970) and Ettinger et al. (2003), indicating that *Naegleria* can actually be found in salt water. The authors identified single cells of *Naegleria* sp. in natural low-salinity water. Water salinity limits the growth of amoebae but does not eliminate them completely; in the Black Sea, amoebae were identified in 30 % of the analysed water samples (Tsvetkova et al. 2004).

The data presented in this study show that the spread of pathogenic amoebae is determined by the combination of several factors. In addition, the data seem to suggest that potentially dangerous FLA and *L*. *pneumophila* may coexist in thermal saline baths used in balneotherapy. This fact should be taken into account while planning and introducing actions aimed at eradicating both groups of these potentially dangerous microorganisms.
